# Thanks for Your Recognition, Boss! a Study of How and When Voice Endorsement Promotes Job Performance and Voice

**DOI:** 10.3389/fpsyg.2021.706501

**Published:** 2021-07-20

**Authors:** Shaoxue Wu, Daisy Mui Hung Kee, Daiheng Li, Dan Ni

**Affiliations:** ^1^School of Management, Universiti Sains Malaysia, Penang, Malaysia; ^2^School of Economics and Management, Beijing Jiaotong University, Beijing, China; ^3^School of Business, Sun Yat-Sen University, Guangzhou, China

**Keywords:** voice endorsement, positive mood, work engagement, voice behavior, voice commitment

## Abstract

Existing studies mainly explore the antecedents and distal outcomes of voice behavior of employees. Less is known about what may occur after supervisors endorse ideas of employees. Based on the conservation of resources theory, we explored how and when voice endorsement affects job performance and voice behavior of employees. With the sample of 444 matched supervisor–subordinate pairs from a large organization, we found that voice endorsement of supervisors positively influences voice behavior and job performance of employees through the mediating effects of positive mood and work engagement. Additionally, we found that the voice commitment of employees strengthens the influence of voice endorsement of supervisors on positive mood of employees. Theoretical and practical implications of these findings are discussed.

## Introduction

Voice, a discretionary or formal expression of ideas, opinions, or suggestions about work-related issues, is crucial in organizational studies (Bashshur and Oc, 2015; He et al., 2020). Employees frequently face situations under which they decide to speak up or stay silent about important work-related issues (Premeaux and Bedeian, 2003). Building on the seminal study of Hirschman (1970), exit, voice, and loyalty over 1,000 studies have examined the impact of voice in organizations. Previous study has explored its effects at all levels of the organization (e.g., individual job attitudes, group innovation, and organizational performance; Klaas et al., 2012). In recent years, several studies have explored the ways of encouraging supervisors to endorse ideas of employees (e.g., Burris, 2012; Lam et al., 2019; Li et al., 2019). Voice endorsement refers to recognition and valuation of voiced ideas of supervisors and the steps they take toward incorporating these endorsed ideas into work practices (Burris, 2012; He et al., 2020).

Various predictors of voice endorsement have been examined in previous studies, ranging from voicer factors (e.g., Whiting et al., 2012; Howell et al., 2015) and supervisor factors (e.g., Fast et al., 2014; Li et al., 2019; Sherf et al., 2019), to voice strategies (e.g., Burris, 2012; Lam et al., 2019). However, there remain significant gaps in the existing voice endorsement literature. First, employees are interested in parties, and their responses can exert important influence over the ultimate effectiveness of voice behavior after it is endorsed by supervisors (Frazier and Bowler, 2015). Voice is an ongoing process, not the one-shot exchange (Takeuchi et al., 2012). However, most voice research has focused on predicting one-time or initial voice behavior without exploring the dynamics inherent in the ongoing need of supervisors and organizations for ideas of employees (Maynes and Podsakoff, 2014; Bashshur and Oc, 2015). Thus, it is essential to examine voice behavior as it evolves over time after supervisors endorse ideas of employees. A long-term perspective in voice research can enrich our understanding of these relationships.

Second, a minimal research has examined subsequent psychological and behavioral responses of employees when their ideas are endorsed by supervisors (Liang et al., 2012; Chen and Hou, 2016). To better understand the effects of voice endorsement, we believed that it is necessary to look at these psychological and behavioral outcomes over time. Thus, the purpose of this study is to develop a theoretical model that unravels the process by which employees respond to voice endorsement in the workplace.

In studying the psychological and behavioral responses of employees following the endorsement of their voice by supervisors, it is valuable to examine the issue from the perspective of the conservation of resources (COR). Although there are calls from several scholars (e.g., Burris, 2012; Lam et al., 2019; He et al., 2020) to focus attention on the long-term perspective, to the best of our knowledge, responses of employees to voice endorsement have not yet been tested in organizational research. Voice endorsement by supervisors gives employees the resources including the positive mental state, which can further affect their behaviors at the workplace.

To fill these gaps and expand our understanding of the impact of voice endorsement of supervisors on the work outcomes of employees, we adopted the COR theory (Hobfoll, 1989). According to this theory, employees feel that increases in precious resources such as identity, self-esteem, and self-confidence will make them feel mentally uplifted, mainly expressed as a supplement of emotional resources (Hunter et al., 2017). Due to the reduced pressure resulting from this, employees can then more easily control their environment and increase their access to certain resources through various other channels (Lin et al., 2019). If the emotional resources of employees are depleted due to unrecognized voice, they cannot obtain other resources necessary to overcome organizational stress and maintain good performance (Miner and Glomb, 2010). When their voices are endorsed, on the other hand, they will have a subjective perception of resource supplement, a sufficiency of personally owned resources, and a sense of security that can, in turn, evolve into a motivational factor affecting the work behaviors and outcomes of employees.

**Figure 1:**
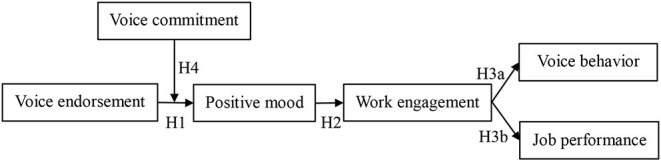
Conceptual model.

Based on the arguments of COR theory, in this study, we believed that when supervisors endorse the voices of employees, the focal employees will have the perception of security and trustworthiness. In this process, the improvement of self-esteem and self-confidence of employees, that is, the increase and supplement of emotional resources (Hunter et al., 2017), can stimulate the positive mood of employees. In conjunction with COR theory, with sufficient resources, employees can focus their energy on work (Gawke et al., 2017). Employees are actively and fully engaged in their work, and then use their resources to identify and solve the problems at work, which in turn brings better performance (Breevaart et al., 2015). At the same time, the resources of self-esteem and self-confidence also help them speak up again to their supervisors about problems at work (King et al., 2019). In addition, employees who are highly committed to their voices have strong senses of identification with their suggestions and are more concerned about the outcomes of their voices (Klein et al., 2014). This study argues that, compared with employees with low voice commitment, employees with high commitment can obtain more emotional resources and have more positive mood resources such as self-esteem and self-confidence when supervisors endorse their voices. Thus, we established the mechanism by which voice endorsement of supervisors affects the voice behavior and job performance of employees through the sequence-mediating roles of the positive mood of employees and work engagement and the moderating role of voice commitment. Figure 1 shows our theoretical framework.

## Theoretical Development and Hypotheses

### Conservation of Resources Theory and Voice Endorsement

Conservation of resources theory suggests that individuals instinctually strive to acquire and maintain their resources (Hunter et al., 2017). Stress and insecurity arise when individuals perceive that they may lose some resources, or have already lost some resources, or have little prospect of acquiring new resources (Kiazad et al., 2015; Carmona-Halty et al., 2019). Individuals perceive stress in three contexts as follows: (1) when there is a threat of loss of available resources, (2) when there is an actual loss of available resources, and (3) when efforts have been made without an actual increase in resources (Lin et al., 2019). In short, the loss of existing resources and the failure to acquire new resources trigger stress responses in individuals, both at the perceptual and objective levels.

According to COR theory (Hobfoll, 1989), resources have the potential to symbolize the status, wealth, and power of an individual in society. Loss of these resources can result in the experience of losing the identity or place of an individual in society, resulting in stressful emotions and insecurity. On this basis, COR theory further suggests that in stressful situations, individuals use existing resources to acquire new resources, thus reducing any loss of resources. In addition, individuals actively build and maintain their current resource reserves to cope with possible resource loss situations (Kim and Kang, 2017; Mehboob and Othman, 2020). COR theory is mainly about conservation, protection, and access to resources.

COR theory provides a theoretical lens for understanding the role of organizations and supervisors play in creating the sense of obligation of employees and promoting their positive attitudes at work (Wayne et al., 2017). Insecurity of employees in an organization is a combination of perceptions of employees of their current resource possession status and the possibility of losing and gaining related to resources in the future (Xia et al., 2019). Based on the COR theory, the voice endorsement of supervisors can promote the maintenance and stability of resources of employees (Kiazad et al., 2015). Voice endorsement of supervisors generates a positive mood among employees, which provides a supplement of emotional resources (Hunter et al., 2017). Due to their perception of controlling their environment (Alarcon, 2011; Lin et al., 2019), employees can then use these resources in boosting their job performance and are more willing to speak up their ideas and opinions to supervisors.

### Voice Endorsement and Positive Mood

Voice is a proactive and prosocial behavior that is cooperative and change-oriented but may bring risks to the voicer (Bashshur and Oc, 2015). It is problem-focused, change-oriented, and constructive (Starzyk et al., 2018). In this study, voice has been viewed as prosocial and improvement-oriented (e.g., McClean et al., 2013; Maynes and Podsakoff, 2014). As a result, voice signifies employee commitment and concern for the organization, and, in turn, supervisors recognize and reward employees who express their voice.

Supervisors play a vital role in the voice process. Supervisors receive voice when their employees want to initiate “change rather than escape from an objectionable state of affairs” (Burris, 2012). Endorsement of supervisors of ideas of employees is an important precursor to make substantive changes in organizational routines or processes (King et al., 2019). When supervisors endorse the voice of employees, employees are expected to have a positive mood. Voice endorsement of supervisors signals safety and trust to the voicer. According to the COR theory, the efforts of employees to acquire, maintain, nurture, and protect their resources are motivated by the basic need to adapt to their environment and sustain (Lin et al., 2019). On the one hand, employees tend to respect and trust their supervisors after their voices are endorsed. On the other hand, it may enhance the sense of pride and self-worth of employees.

A positive mood is a mediator in our model. It entails enduring experiences of pleasant (e.g., excited) affective states (Warr et al., 1983; Watson et al., 1988). Although emotions are short-lived and intense reactions to specific events, moods typically have a longer duration (e.g., several days, weeks, or months) and are more generalized in focus (Brief and Weiss, 2002; Barsade and Gibson, 2007). After the supervisor endorses the voices of employees, employees can perceive their interpersonal support of supervisors. They feel a level of comfort and trust, which is a supplement to their resources. This leads to an internal and lasting positive emotion in employees, resulting from the increased resources and good feelings they experience when their supervisors accept their voice. To summarize the above, we hypothesized the following:

*Hypothesis 1: Voice endorsement of supervisors is positively related to the positive mood of employees*.

### Positive Mood and Work Engagement

Work engagement refers to a “psychological identification with the job and seeing performance as a reflection of one's values” (Salanova et al., 2005). It is defined as a positive, fulfilling, work-related state of mind characterized by vigor, dedication, and absorption (González-Romá and Bakker, 2002). When employees have a positive mood, they tend to have high energy and mental resilience levels. According to COR theory, these adequate resources can enhance intrinsic motivation and investment of employees and improve the work identity of employees (Xu et al., 2015). It has been pointed out in previous study that employees with a positive mood can be strongly involved in their work and can experience a sense of significance, enthusiasm, inspiration, pride, and challenge (Costa et al., 2014). When employees invest more time and energy into their work, they will have better performance, reflecting their values (Miner and Glomb, 2010).

Furthermore, based on the COR theory, employees with a positive mood toward work give them adequate resources to take control of the work process (Chatterjee and Hambrick, 2011). Some studies (Miner and Glomb, 2010; Madrid et al., 2014) have found that the positive mood of employees is associated with improved job identification and motivation. In this state, employees can remain resilient even in the face of challenges and difficulties. Therefore, employees with a positive mood are motivated to get involved in their job and focus on actions that increase their engagement in their work. The above discussion led us to propose the following hypothesis:

*Hypothesis 2: Positive mood of employees is positively related to their work engagement*.

### Work Engagement and Outcomes

The work engagement of employees can positively influence their work outcomes (i.e., voice behavior and job performance). First, when employees are fully engaged in their work, they devote their resources to identifying and facing problems at work (Sekhar et al., 2018). The emotional resources that employees have, such as self-esteem and self-confidence, help them speak up to their supervisors. Although the voice of employees is a risky behavior (Bashshur and Oc, 2015), the voice endorsement can alleviate the stress and fear of doing so again on subsequent occasions. Based on the COR theory, when employees are in a state of stress or fear, it will cause the depletion of their resources (Fatima et al., 2018). With the reduction in stress associated with risky behavior, the resources that employees put into their work will not be lost. More resources can be applied to identify work problems and speak up to their supervisors. Therefore, highly engaged employees who have sufficient emotional resources at work are more likely to speak up.

Second, when employees are engaged in their work, they constantly devote resources to asking, analyzing, and solving problems (Lan et al., 2020). Through this process, employees can effectively develop their work skills, which improve their job performance. By investing resources, employees demonstrate high energy and good psychological resilience at work (Shin et al., 2012). They can concentrate on their work and take up challenges. Adequate emotional resources make employees more proactive and less affected by the negative emotions of job demands, such as workload, time pressure, and role conflict (Zhang et al., 2018). Engaged employees are passionate about their work, and they have a strong sense of identity and pride in their work (Xu et al., 2015). With the input of resources, these employees can develop beneficial skills, and their job performance increases with the high level of work engagement (Qin et al., 2018). Previous studies of work engagement based on the COR theory (Breevaart et al., 2015; Sekhar et al., 2018) have also found that work engagement is positively associated with job performance. Thus, highly engaged employees will have better job performance accompanied by the input of resources and energy. The above discussion is summarized in the following hypotheses:

*Hypothesis 3a: Work engagement of employees is positively related to their voice behavior*.

*Hypothesis 3b: Work engagement of employees is positively related to their job performance*.

### The Moderating Effect of Voice Commitment

Voice commitment, similar to organizational commitment, refers to the strength of identification of employees and participation in their voice (Lapointe and Vandenberghe, 2018). Some voice commitment studies have tried to find ways of improving how employees feel about their voice so that these employees would become more committed to their organizations (Klein et al., 2014). Employees with high voice commitment have a pretty strong sense of identification with and belonging to their previous voice.

Specifically, employees who have a high commitment to their voice are concerned about their voice and sensitive to the results of their voice (Klein et al., 2014). In line with the COR theory, when supervisors endorse the voice of employees, the latter is more likely to derive self-confidence and self-worth (Guzman and Espejo, 2019). The positive relationship between voice endorsement and positive mood will be enhanced. However, if supervisors do not endorse the voices of employees, employees tend to perceive their value as unappreciated by their supervisors and experience poor mood (King et al., 2019). Therefore, high voice commitment may reinforce the positive relationship between voice endorsement and positive mood. The above discussion is summarized in the following hypothesis:

*Hypothesis 4: Voice commitment may strengthen the effects of voice endorsement on the positive mood of employees*.

## Methods

### Sample and Procedure

The participants in this study were employees and their immediate supervisors working in a manufacturing company in China. Before conducting the surveys, we interviewed employees, who suggested that voice endorsement was common in this company, making it suitable for this study. The participants, comprising 1,208 employees and 392 immediate supervisors, took part voluntarily and were assured that their responses would be kept anonymous and confidential. Using the list of names supplied by the human resources department of organizations, we used a matched four-digit code to identify each employee and their supervisor.

To reduce the potential common method bias, we collected four waves of data. Each of the four waves was separated by 1 month. At Time 1, employees reported voice endorsement, voice commitment, and control variables. At Time 2, employees reported their positive mood. At Time 3, we asked employees to report the variables of work engagement. At Time 4, supervisors reported the voice behavior and job performance of employees.

At Time 1, we distributed 1,208 questionnaires to employees and received 1,042 completed questionnaires. At Time 2, we distributed 1,042 questionnaires to those employees who had returned their questionnaires at Time 1 and received 745 completed questionnaires. At Time 3, we distributed questionnaires to those 745 employees who had submitted valid questionnaires at Time 2, and 639 employees returned their completed questionnaires. At Time 4, supervisor questionnaires were distributed to the supervisors of these 639 employees. Following these four waves of data collection, we obtained 444 supervisor–employee pairs of valid data by matching.

Overall, 58.11% of the participants were males, and the average age of participants was 34.84 years. Their average organizational tenure was 4.79 years.

### Measures

Since all the measures were originally constructed in English, we used the back-translation method to translate all items. We used a seven-point Likert-type scale (i.e., 1 = completely disagree to 7 = completely agree) for all the measures.

#### Voice Endorsement

Voice endorsement of supervisors was assessed using the five-item scale of Burris (2012). A sample item is, “I think this employee's comments should be implemented” (α = 0.93).

#### Positive Mood

We used a five-item scale of Mackinnon et al. (1999) to assess the positive mood of employees. A sample item is, “I am inspired with my supervisor” (α = 0.92).

#### Work Engagement

We used the nine-item scale of Schaufeli et al. (2006) to measure the work engagement of employees. A sample item is, “At my work, I feel bursting with energy” (α = 0.98).

#### Voice Behavior

Supervisors rated the voice behavior of employees using a four-item scale from Van Dyne and LePine (1998). A sample item is, “This employee developed and made recommendations concerning issues that affect the organization” (α = 0.98).

#### Job Performance

Job performance of employees was evaluated by their supervisors using the five-item job performance scale of Tsui et al. (1997). A sample item is, “This employee adequately fulfills his/her job responsibilities” (α = 0.96).

#### Voice Commitment

Employees reported their voice commitment using a four-item scale by revising items from Klein et al. (2014). A sample item is, “I am committed to my suggestions” (α = 0.96).

#### Control Variables

Following previous studies on voice behavior and job performance (Belenky et al., 1997; Ng and Feldman, 2010; Roth et al., 2012; Tangirala et al., 2013), we controlled for gender, age, organizational tenure of employees, and the level of education to rule out the possibility that those demographics might influence the outcomes. Gender may influence voice behavior, as men face fewer psychological barriers when sticking to their opinions (Belenky et al., 1997). And researchers in organizational behavior have found gender differences in measures of job performance (Roth et al., 2012). More experienced employees (as reflected in their age or organizational tenure) may be more familiar with operations that can enhance their ability to speak up (Tangirala et al., 2013). In addition, organizational tenure of employees may influence their job performance (Ng and Feldman, 2010). Similarly, employees with a higher level of education may be more confident in their voice behaviors (Tangirala et al., 2013).

Furthermore, some studies of human capital have pointed out that the accumulation of experience can help employees get more resources, stronger working ability, and better work outcomes (Wei, 2015; Bernerth and Aguinis, 2016; Raffiee and Coff, 2016). Therefore, the characteristics of employees, such as age, organizational tenure, and level of education, may influence their work behaviors and outcomes (i.e., voice behavior and job performance).

## Results

### Descriptive Analyses

Table 1 shows the descriptive statistics and correlations among the variables.

**Table 1:** Descriptive statistics and correlations among study variables.

**Variable**	**Mean**	**SD**	**1**	**2**	**3**	**4**	**5**	**6**	**7**	**8**	**9**
Gender	1.42	0.49									
Age	34.84	6.33	−0.02								
Education	5.33	0.84	−0.02	0.04							
Organizational tenure	4.79	4.16	−0.03	0.58^***^	0.05						
Voice endorsement	5.02	1.43	−0.05	−0.02	0.01	−0.09					
Voice commitment	5.29	1.33	0.01	0.02	0.10^*^	−0.03	0.28^***^				
Positive mood	5.27	1.25	−0.05	−0.08	0.02	−0.06	0.37^***^	0.06			
Work engagement	5.40	1.20	−0.01	0.00	−0.07	−0.02	0.28^***^	0.08	0.45^***^		
Voice behavior	5.35	1.43	0.00	−0.01	−0.02	−0.02	0.32^***^	0.13^**^	0.31^***^	0.44^***^	
Job performance	5.11	1.29	0.00	0.01	−0.10^*^	−0.08	0.28^***^	0.15^**^	0.37^***^	0.44^***^	0.43^***^

*n = 444. Gender: 1 = male and 2 = female; education: 1 = primary school, 2 = junior high school, 3 = high school, 4 = college degree, 5 = bachelor's degree, 6 = master's degree, and 7 = doctor's degree; organizational tenure was measured as the number of years in the current company*.

* *p < 0.05;*

** *p < 0.01;*

*** *p < 0.001 (two-tailed test)*.

### Confirmatory Factor Analysis

Before testing our hypotheses, we conducted the confirmatory factor analysis (CFA) of the study variables with Mplus 8.0 and the manufacturer's location for Mplus 8.0 is Los Angeles, California (Muthén and Muthén, 2010). As shown in Table 2, the results showed that the hypothesized six-factor model [χ^2^_(449)_ = 1,790.51, *p* < 0.001; root mean square error of approximation (RMSEA) = 0.08, standardized root mean square residual (SRMR) = 0.03, comparative fit index (CFI) = 0.93, Tucker-Lewis index (TLI) = 0.92] fits the data well, and this model fits the data significantly better than the other alternative models. Thus, the results indicated that the focal variables had discriminant validity.

**Table 2:** Confirmatory factor analysis.

**Model**	**χ^2^**	***df***	**Δχ^2^(*df*)**	**RMSEA**	**SRMR**	**CFI**	**TLI**
*The hypothesized six-factor model*	1790.51	449		0.08	0.03	0.93	0.92
*The five-factor models*							
Voice behavior and job performance as a factor	4037.05	454	2246.54^***^	0.13	0.11	0.82	0.80
Voice commitment and positive mood as a factor	3544.69	454	1754.18^***^	0.12	0.09	0.84	0.83
*The four-factor models*							
Voice behavior and job performance as a factor; voice commitment and positive mood as a factor	5781.02	458	3990.51^***^	0.16	0.20	0.73	0.70
Work engagement, voice behavior, and job performance as a factor	6447.76	458	4657.25^***^	0.17	0.13	0.69	0.67
*The three-factor model*							
Voice commitment and positive mood as a factor; work engagement, voice behavior, and job performance as a factor	8189.67	461	6399.16^***^	0.19	0.21	0.60	0.57
*The two-factor model*							
Voice commitment, and positive mood, work engagement, voice behavior, and job performance as a factor	9848.31	463	8057.80^***^	0.21	0.18	0.52	0.48
*The single-factor model*							
All variables as a factor	11418.68	464	9628.17^***^	0.23	0.20	0.44	0.40

*** *p < 0.001 (two-tailed test)*.

### Hypotheses Testing

We conducted the path analyses using Mplus 8.0 (Muthén and Muthén, 2010). In all analyses, we grand-mean centered the independent variable, the moderator, and control variables. As shown in Table 3, voice endorsement is positively related to the positive mood of an employee (γ = 0.35, SE = 0.05, and *p* < 0.001), and positive mood of an employee was positively and significantly related to work engagement (γ = 0.39, SE = 0.05, and *p* < 0.001), supporting Hypotheses 1 and 2. Meanwhile, the work engagement of an employee was positively related to voice behavior (γ = 0.41, SE = 0.07, and *p* < 0.001) and job performance (γ = 0.33, SE = 0.06, *p* < 0.001). Thus, Hypotheses 3a and 3b were supported by the data.

**Table 3:** Results of path analyses.

**Variable**	**Positive mood**	**Work engagement**	**Voice behavior**	**Job performance**
Gender	−0.11 (0.11)	0.03 (0.10)	0.05 (0.12)	0.03 (0.11)
Age	−0.02 (0.01)	0.01 (0.01)	−0.00 (0.01)	0.02 (0.01)
Education	0.05 (0.07)	−0.12^*^ (0.06)	−0.01 (0.08)	−0.14^*^ (0.07)
Organizational tenure	−0.01 (0.02)	0.00 (0.01)	0.01 (0.02)	−0.03^*^ (0.02)
Voice endorsement	0.35^***^ (0.05)	0.11^*^ (0.05)	0.18^**^ (0.05)	0.09 (0.05)
Voice commitment	0.05 (0.05)	0.03 (0.05)	0.07 (0.05)	0.09^*^ (0.04)
Voice endorsement × Voice commitment	0.16^***^ (0.03)	0.01 (0.03)	0.03 (0.03)	0.01 (0.03)
Positive mood		0.39^***^ (0.05)	0.09 (0.07)	0.20^***^ (0.06)
Work engagement			0.41^***^ (0.07)	0.33^***^ (0.06)
Pseudo-*R*^2^	21.60%	22.26%	24.29%	25.05%

*n = 444. Numbers in parentheses are SE of coefficients*.

* *p < 0.05;*

** *p < 0.01;*

*** *p < 0.001 (two-tailed test)*.

**Figure 2:**
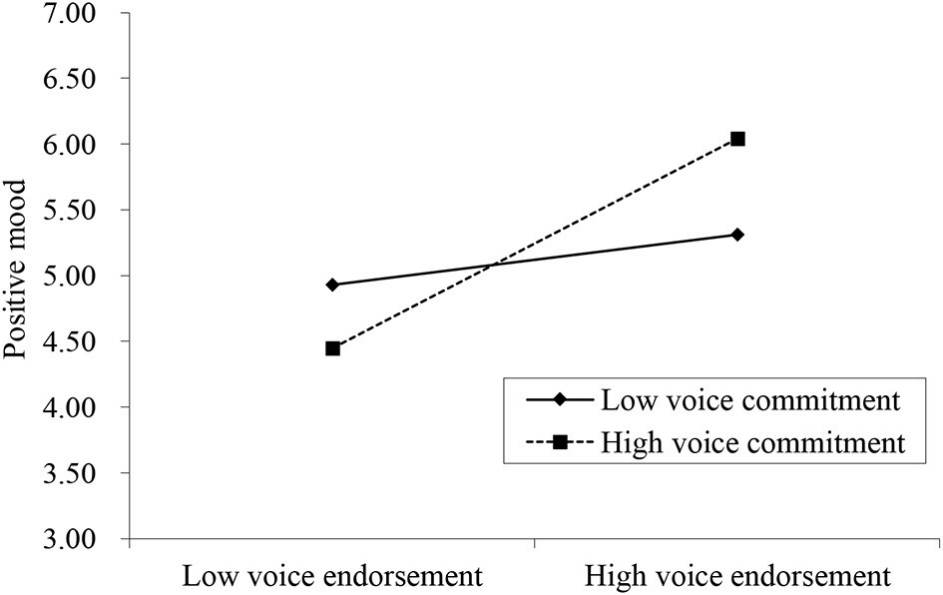
The moderation effect of voice commitment on the relationship between voice endorsement and positive mood.

Hypothesis 4 proposed that voice commitment moderates the relationship between voice endorsement and positive mood. The results showed that the coefficient of the interaction term was significant (γ = 0.16, SE = 0.03, and *p* < 0.001). Figure 2 shows a plot of this interaction effect at conditional values of voice commitment (1 SD above and below the mean). When we conducted a simple slope analysis, as recommended by Preacher et al. (2006), the results demonstrated that the positive relationship between voice endorsement and positive mood was significant at a higher level (1 SD above the mean) of voice commitment (simple slope = 0.56, *t* = 10.95, and *p* < 0.001) but the effect became reduced at a lower level (1 SD below the mean) of voice commitment (simple slope = 0.13, *t* = 2.80, and *p* < 0.01). Thus, Hypothesis 4 was supported.

## Discussion

Based on the COR theory, we advanced and examined a model about how the voice endorsement of supervisors affects the voice behavior and job performance of employees through the mediating roles of the positive mood and work engagement of employees, and how voice commitment moderated the effect of voice endorsement of supervisors on the positive mood of employees.

This study found that when supervisors endorse the voices of employees, employees will have a subjective perception of resource supplement, a sufficiency of personally owned resources, and a sense of security. Sufficient energy makes employees generate a positive mood. These employees can then engage their resources at work and finally boost their job performance and continue to voice their ideas to supervisors. In addition, the results showed that compared with employees with low voice commitment, employees with high commitment can better supplement their emotional resources when supervisors endorse their voices. Existing voice literature mainly explores factors leading to voice endorsement (e.g., Burris, 2012; Lam et al., 2019; Li et al., 2019), and less is known about what occurs on employees after supervisors endorse their ideas (Frazier and Bowler, 2015; Chen and Hou, 2016). We enriched the voice research by examining when and how the voice endorsement of supervisors can enhance the voice behavior of employees and job performance. Furthermore, we also displayed the role that COR plays in the consequences of voice endorsement.

### Theoretical Implications

This study contributes to the literature in the following three ways: first, we contributed to the COR theory by enriching our understanding of how employees respond to voice endorsement. Although the critical role of voice endorsement in unpacking employee subsequent behavioral reactions is noted (King et al., 2019; He et al., 2020), COR theory does not specify whether and how voice endorsement influences these outcomes. Existing COR theory literature mainly focuses on factors that can generate the first-time voices of employees (Ng and Feldman, 2012). Some researchers have also pointed out that voice is not one-time behavior, and supervisors need the ideas of employees continuously (Maynes and Podsakoff, 2014; Bashshur and Oc, 2015). However, the existing empirical research on the effect of voice endorsement of supervisors for employees to make suggestions again is pretty rare. King et al. (2019) established a model using social exchange theory with non-endorsement of suggestions as to the antecedents. This study contributes to this gap by examining how the voice endorsement of supervisors influences the voice behavior of employees.

Second, we extended the voice research by examining voice as an ongoing process and from a long-term perspective. Many scholarly efforts (e.g., Burris et al., 2013; Bashshur and Oc, 2015; Aryee et al., 2017) have been employed to examine the antecedents and distal outcomes of voice. However, what these studies lack is a specific focus on those instances when the ideas of employees are endorsed by supervisors and the subsequent effects. Most voice research has focused on the prediction of one-time, initial voice behavior (Maynes and Podsakoff, 2014; Bashshur and Oc, 2015). This study is essential to fill this gap by examining persistent voice behavior.

Third, we shed light on the psychological and behavioral responses of employees after supervisors have endorsed their ideas. Voice is challenging in general (Van Dyne and LePine, 1998) and can be challenging for supervisors (Tangirala and Ramanujam, 2008). Considering these risks, employees may sometimes feel reluctant to express their voice (Milliken et al., 2003). However, when they have previous experience of their voice being endorsed by supervisors, employees can develop positive psychological and behavioral mechanisms over time. This study found that the psychological state of employees will be improved after their supervisors endorse their voice. They also become more likely to make their voice heard again. This study helps scholars to understand how the voice endorsement of supervisors affects psychology and behaviors (i.e., by promoting positive mood and work engagement) of employees at the workplace.

### Practical Implications

Our results have several practical implications. First, this study found that voice endorsement has a significant impact on the work outcomes of employees. Therefore, supervisors should give a thoughtful consideration to the voices of employees, take a positive attitude to adopt good suggestions, and explain to employees the reasons why some suggestions may be inappropriate. Supervisors who adopt a reasonable approach to the voice of employees can bring about certain benefits for both employees and the organization. In addition, since employees whose voices are endorsed will engage more in their work and perform better in their tasks, supervisors can assign them to complete more challenging tasks. Thus, voice endorsement both exercises the workability of employees and enhances the efficiency of the work team.

Second, our results showed that the positive mood of employees leads to desirable work outcomes. This study also revealed that the emotional resources that employees get from their supervisors can benefit their work. These findings suggest that supervisors need to use suitable approaches to bring emotional resources to employees and help them improve positive emotions. For example, when employees speak up for the work, supervisors should appreciate and recognize the reasonable ideas of employees. Besides, organizations may provide training to employees on how to regulate the emotions themselves and grasp the promotion of positive mood in the workplace.

Third, if supervisors endorse the voices of employees, employees need to grasp this opportunity. They should engage more time in challenging or innovative tasks to improve their workability. In addition, employees should voice to or communicate with their supervisors if they have new ideas and suggestions while handling these tasks. This ongoing process of suggesting ideas to the supervisor can help employees develop their voice into a habitual behavior at work. Habitual voice behavior facilitates employees to enhance communication with supervisors so that the supervisors know their work in a timely manner. In this ongoing process, the voices of employees at the organizational level also help the organization identify problems and address them.

### Limitations and Future Research

There are several limitations to this study. First, this study only explored the mediating mechanisms of positive mood and work engagement. Other mediating paths, such as self-confidence and perceived obligation, may also impact the work outcomes of employees. For example, in the study by Guzman and Espejo (2019), it was pointed out that the endorsement of their voice by supervisors helps employees improve their self-confidence in carrying out subsequent work tasks. In addition, we also believed that there are double sides to voice endorsement: on the one hand, it promotes positive emotions among employees; on the other hand, it possibly increases pressure on them to reciprocate their support obligations of supervisors (King et al., 2019). Thus, future researchers should explore other mediating mechanisms in depth.

Second, our samples were from employees working in a collectivist culture, and their beliefs might differ from those of employees working in individualistic cultures. In the collectivist Chinese culture (Hofstede, 1984), employees are particularly concerned about whether their voice is supported and endorsed by their supervisors (Lam et al., 2019). Voice endorsement in this culture may thus generate stronger emotional responses and greater work efforts than individualistic cultures. Therefore, in order to increase the generalizability of the results, future researchers need to collect data from different cultural backgrounds.

## Data Availability Statement

The raw data supporting the conclusions of this article will be made available by the authors, without undue reservation.

## Ethics Statement

The studies involving human participants were reviewed and approved by Beijing Jiaotong University. The patients/participants provided their written informed consent to participate in this study. Written informed consent was obtained from the individual(s) for the publication of any potentially identifiable images or data included in this article.

## Author Contributions

SW wrote theories and hypotheses. DK improved the research idea, theory, and conceptual model. DL was responsible for data analysis. DN was responsible for data collection. All authors contributed to the article and approved the submitted version.

## Conflict of Interest

The authors declare that the research was conducted in the absence of any commercial or financial relationships that could be construed as a potential conflict of interest.
